# Feeding Sheep Cobalt and Oregano Essential Oil Alone or in Combination on Ruminal Nutrient Digestibility, Fermentation, and Fiber Digestion Combined With Scanning Electron Microscopy

**DOI:** 10.3389/fvets.2021.639432

**Published:** 2021-06-09

**Authors:** Ting Jiao, Jianping Wu, David P. Casper, Delmer I. Davis, Michael A. Brown, Shengguo Zhao, Jianyong Liang, Zhaomin Lei, Bill Holloway

**Affiliations:** ^1^College of Grassland Science, Key Laboratory of Grassland Ecosystem, Gansu Agricultural University, Lanzhou, China; ^2^College of Animal Science and Technology, Gansu Agricultural University, Lanzhou, China; ^3^Animal Husbandry, Pasture and Green Agriculture Institute, Gansu Academy of Agricultural Sciences, Lanzhou, China; ^4^Casper's Calf Ranch, LLC, Freeport, IL, United States; ^5^Department of Animal Sciences, North Carolina Agricultural and Technical State University, Greensboro, NC, United States; ^6^Ralco Nutrition. Inc., Abilene, TX, United States; ^7^B&B Research & Development, El Reno, OK, United States; ^8^Ralco Nutrition Inc., Uvalde, TX, United States

**Keywords:** cobalt, essential oil, digestibility, fiber structure, sheep, scanning electron microscopy

## Abstract

The feeding of Co lactate (Co), an essential oil blend (EO; oregano), or a combination of Co and EO (EOC) may improve nutrient digestion of corn silage-based rations. In four separate studies, Co, EO, or EOC was fed at 0, 4, and 7 g/days to nine rumen fistulated rams arranged in a replicated 3 × 3 Latin square design. The fourth study evaluated the carrier at 0, 4, and 7 g/day. In each ram, fresh ensiled corn silage, leaf, and husk were placed in individual nylon bags inserted through the ruminal cannula and removed after 48 h. Rams fed increasing carrier rates demonstrated similar (*P* > 0.10) nutrient digestibilities and ruminal pH and volatile fatty acid concentrations. Feeding Co at 4 and 7 g/day increased (*P* < 0.05) digestibility of DM (59.4, 63.9, and 62.4% for 0, 4, and 7 g/day, respectively), NDF (59.4, 63.9, and 62.4%), and hemicellulose (HC; 56.2, 63.6, and 65.9%) compared with rams fed 0 g/day, while CP digestibility (46.4, 49.9, and 57.8%) was improved (*P* < 0.05) in rams fed 7 g/day compared with those fed 0 and 4 g/day. Rams fed 4 g/day EO digested greater (*P* < 0.05) HC (64.1, 71.4, and 69.1%) than rams fed 0 g/day, while rams fed 7 g/day were intermediate and similar (*P* > 0.10). Rams fed the EOC combination at 4 and 7 g/day demonstrated greater (*P* < 0.05) digestibilities of DM (57.7, 60.0, and 60.0%), NDF (21.4, 28.8, and 27.7%), and ADF (24.3, 33.3, and 34.4%) than rams fed 0 g/day. The SEM and SM techniques visually demonstrated minor evidence of husk and leaf digestibility in rams across the three experiments when fed 0 g/day of Co, EO, or EOC; rams fed 4 g/day of Co, EO, or EOC exhibited varying visual signs of leaf digestion with some palisade tissue, spongy tissue, and whole vein structure remaining, while in rams fed 7 g/day, only the vein structure remained. Results demonstrated that feeding Co, EO, or EOC at 4 or 7 g/day enhanced ruminal nutrient digestion and fermentation parameters, which was visually confirmed via SEM and SM.

## Introduction

China has a large agricultural economy with nearly 400 million ha of grassland, but overgrazing has resulted in significant amounts of confinement feeding using poorly digestible mature forages and crop residues, i.e., corn stalks, straw, etc. Various technologies exist to enhance nutrient and fiber digestibility among forage and crop residues having a wide digestibility range, but two promising technologies are feeding cobalt lactate (Co) and essential oils (EOs).

Co has been shown to improve fiber digestion, both in *in vitro* (1, 2) and *in vivo* studies (3–5). Oregano oil is a natural-plant-extracted EO that has a Generally Regarded as Safe (GRAS) status for livestock consumption (6–8). Oregano is reported to have antifungal, antiviral, and Gram-positive and Gram-negative bactericidal and bacteriostatic effects (8, 9). Initial EO ruminal fermentation studies demonstrated an inhibition of gas production (10, 11), and later studies reported altered ruminal fermentation by improving protein metabolism, volatile fatty acid (VFA) production, fiber digestion, and microbial community alteration (8, 12, 13), which altered milk composition (14–16).

A number of research studies conducted by our team has demonstrated improved calf growth when including EO to the milk replacer fed to calves or a blend of Co and EO (Rum-A-Fresh International or Stay Strong Domestically, Ralco Inc., Marshall, MN) fed in the calf starter or ration to growing calves and bulls (7, 17–19) and lactating dairy cows (20). These *in vitro* and *in vivo* trials demonstrated that feeding the Co and EO blend shifted ruminal fermentation to more total VFA and molar propionate percentages while reducing methane emissions and altering the microbial community, which led to improved dry matter, protein, and fiber digestion. However, in order to determine the exact mechanism of action, the individual components along with the combination were evaluated in a mechanistic study to identify specific responses to each component and identify the potential synergistic mechanisms.

Our current research program is focused on improving forage and crop residue utilization for enhancing ruminal N and fiber (energy) utilization by sheep. Most Co and EO (oregano) research projects have used fixed inclusion rates, and little is known regarding responses based on varying the inclusion rates combined with possible synergistic mechanisms among these technologies. For example, Froehlich et al. (7) reported that EO feeding rates used in an earlier work may have been too high to elucidate growth responses, and those high EO feeding rates may have been detrimental to growth performance. Froehlich et al. (7) demonstrated improved growth rates when feeding the lowest (2.5 g/day) EO inclusion rate compared to higher EO feeding rates. The study hypothesis was that sheep fed Co, EO, and/or EOC (combination) could potentially benefit alone or in combination from application of these fiber-digesting-enhancing technologies for improving ruminal nutrient digestion and fermentation, and these ruminal responses may be influenced by feeding rate. Therefore, the experimental objectives were as follows: (1) to measure nutrient digestion and ruminal fermentation characteristics in rams when fed increasing rates of Co and oregano EO alone or in combination (EOC) and (2) to elucidate the beneficial effects of feeding these additives alone or in combination for improving ruminal nutrient (fiber) digestibility. This study was designed to be a mechanism study in contrast to an animal performance study. The novel and unique use of a scanning electron microscope (SEM) and stereoscopic microscopy (SM) in digestion studies has not been conducted before, to our knowledge, which contributes to this study's uniqueness. The use of SEM and SM techniques does not allow for analyzing large numbers of samples from animal performance trials. However, the use of SEM and SM allows for visual observations to confirm findings of digestion.

## Materials and Methods

### Experimental Treatments

All experiments were conducted according to the Standards for the Care and Use of Research Animals (21) at Lintao Dairy and Animal Research Farm of Gansu Agricultural University, China or at Gansu Agricultural University, Lanzhou, China. All procedures were approved by the Institutional Animal Care and Use Committee of Gansu Agricultural University. The three additives evaluated as individual experiments were the following: (1) Co, 0.1425% cobalt lactate + 97% carrier + 2.8575% herbal package; (2) oregano EO, 1.13% essential oil (primarily microfused oregano oil particles <5 μm in diameter and uniform in size + small amount of olive oil) + 97% carrier + 1.87% herbal package; and (3) EOC, 0.1425% cobalt lactate + 1.13% oregano essential oil + 97% carrier + 1.7275% herbal package. The herbal carrier (CARR) product was composed of 75% zeolite (clinoptilolite) + 12% limestone + 10% diatomaceous earth and a herbal package that included small amounts of lactic acid, kelp, roughage products, chicory root, red pepper, fenugreek flavor extract, anise oil, cloves, saccharin sodium, and guar gum. The three additives and CARR were manufactured, packaged, shipped, and donated for the research project by the Animal Health Division of Ralco, Inc. (Marshall, MN, USA). Four independent similar experiments (three additives and CARR) were conducted evaluating the three feed additives (Co, EO, and EOC) and CARR, which were fed at 0 (control), 4, and 7 g/day using a thrice replicated 3 × 3 Lain square design having 21-day periods, with the last 10 days of each experimental period including incubation of corn silage, husk, and leaves samples placed in individual nylon bags (i.e., *in situ*). The 7 g/day inclusion rate was based on a company (Ralco, Inc.)-recommended EOC feeding rate of 28 g/day for lactating dairy cows scaled down to a sheep's average body weight. A misunderstanding/miscommunication between all coauthors on the experimental design resulted in the 0 g/day feeding rate for the Co, EO, and EOC experiments not including the product carrier (CARR) in 0 g/day treatment; therefore, the CARR (fourth) experiment was conducted at a later date at Gansu Agricultural University, Lanzhou, China to demonstrate that the CARR was not influencing the experimental results in the first three experiments because repeating the entire experimental series was not possible.

The total mixed ration (TMR) was formulated to meet the nutrient requirements of a ram weighing 55 kg and gaining 50 g/day according to the mutton sheep breeding standards (NY/T816-2004) of the Agricultural Industry Standard of the People's Republic of China. The forage to concentrate ratio was ~70:30 (1.6 kg/day maize straw silage and concentrate supplement). The ingredient and nutrient compositions of the TMR are given in Table 1. The TMR was mixed daily (Animal Husbandry Machinery Co., Ltd., Hebei, China) and fed twice daily at 9:00 a.m. and 5:00 p.m. with *ad libitum* access to water.

**Table 1:** Ingredient composition and nutrient concentration of the experimental diet.

**Ingredient**	**(% As Is)**	**Nutrient**	**% of DM**
Corn straw silage	72.96	DM (%)	36.2
Corn	14.41	DE (MJ/kg)	14.1
Bran	4.00	ME (MJ/kg)	11.5
Rapeseed meal	3.59	CP (%)	14.7
Cottonseed meal	3.86	Ca (%)	0.76
1% Premix	0.29	P (%)	0.65
Calcium bicarbonate	0.31		
Mineral meal	0.31		
Salt	0.28		
Total	100.00		

### Animals and Management

Three months prior to the start of the experiment, nine healthy German Merino sheep ♂ × Lintao local hybrid ♀ second-generation brucellosis-negative rams were surgically fitted with a permanent rumen fistula (China Agricultural University, Beijing, China). After surgery, the rams were fed a supplement formulated for the prevention of parasites and diseases while recovering and maintaining body condition during the postsurgical healing period. Rams averaged 53.7 kg body weight (BW) with an average rectal temperature of 39.8°C during postsurgical recovery. At the initiation of the experiment, the fistulated rams were blocked by BW and age and randomly assigned to one of three Latin squares having three treatments of each individual additive fed at 0, 4, and 7 g/day, while being housed in a shade-covered, open-sided, naturally ventilated pens. Dry matter intake was 1.02 ± 0.05 kg/day across three experiments (Co, Eo, and EOC), but individual dry matter intakes (DMIs) were not recorded. Thao et al. (22) reported no EO impacts on DMI. For the CARR experiment, three rams with permanent rumen cannula, using the same surgical procedures as described above, weighing ~50 kg, were arranged in a 3 × 3 Latin square following the same experimental protocol.

### Sampling

Ensiled corn silage was sampled weekly and divided into three subsamples: the first subsample was used for DM concentration; the second subsample (100 g) was immediately taken to the laboratory and blended (QE, Zhejiang, China) for ~60–80 s to be used for measuring ruminal nutrient digestion after weighing into nylon bags (*in situ*); and the third subsample was stored at −20°C for later nutrient analyses. The average corn silage nutrient concentrations across all experiments was DM of 93.0 ± 0.20%, CP of 5.66 ± 0.10%, NDF of 56.0 ± 0.83%, and ADF of 28.2 ± 2.07% on a DM basis.

### Ruminal Digestion of Corn Silage, Leaf, and Husk Tissue

At the beginning of each sampling period, three individual nylon bags (400 mesh, 6.5 × 9.5 cm tied with nylon cord) containing 4 g of thoroughly ground fresh corn silage sample were inserted through the ruminal fistula to measure 48-h nutrient digestion. Each ram received three individually labeled rumen nylon bags in each of the three time periods for each additive treatment amounts (0, 4, and 7 g/day) and one empty bag for correction of residual contamination. At the same specified time of nylon bag insertion for 48-h nutrient digestibility, the treatment feeding rate of 0, 4, or 7 g/day of the specific experimental additive (Co, EO, or EOC) were weighed and wrapped in paper in advance and then daily placed directly through the rumen fistula into different ruminal areas to ensure that the specific additive was completely consumed. Thus, consistent with a replicated 3 × 3 Latin square, each ram in each square received each additive amount in the three different Latin square periods. Thus, each treatment additive and its requisite feeding amount resulted in three rams/treatment for a specific time period (i.e., 1, 2, or 3). After 48 h fermentation, all nylon bags attached via a nylon cord were removed and washed gently with 39°C running water until the rinse water was clear. Rinsed bags were then placed in a 65°C drying oven and dried to a constant weight. This nutrient digestibility procedure was performed separately for each of the individual additives (Co, EO, EOC, and CARR) across the four experiments.

Additionally, leaf and husk samples were sourced from the ensiled corn silage that were of sufficient size for cutting into 1 × 1-cm lots. Leaf and husks were selected not only due to their high fiber content but also due to their thin fiber structure for light to illuminate for microscopic scanning. One leaf and husk sample were placed in a separate nylon bag, sealed, and placed through the ruminal fistula into the rumen at the same time as the corn silage nutrient digestibility samples. After 48 h of ruminal digestion and fermentation, the undigested and digested corn silage tissues (leaf and husk) were removed from the rumen, then removed from the nylon bags and placed and fixed to a glass slide containing precooling 2.5% glutaraldehyde solution, then cut into the size of 0.5-cm^2^ area with scissors. These leaf and husk corn silage samples were treated using the same procedures and then prepared for SEM and SM via the procedures of Jiao et al. (23).

### Rumen Fluid Collection

After removal of the nylon bags, containing corn silage and leaf and husk, from the rumen at 48 h, subsequently 50 ml of rumen fluid was extracted from each ram during each period and filtered through four layers of cheese cloth into a clean sampling container. The rumen fluid pH was immediately recorded using a pH meter (HI98103, Shanghai, China) with a glass electrode. The rumen fluid was then preserved by freezing in a cryopreservation fridge (−20°C). Samples were thawed and analyzed for NH_3_-N (colorimetric method described below) and VFA concentrations.

### Laboratory Analysis

All feed and *in situ* residue samples were dried at 65°C (AOAC, 2019; 930.15) in a forced-air oven (DHG - 9240A, Shanghai, China) for 6–8 h to a constant weight and ground through a 1-mm screen and analyzed for CP (990.03), ADF (973.18), and NDF (2002.04) using Association of Official Agricultural Chemists (AOAC) International standard laboratory procedures (2016).

After 48 h of rumen fermentation, the nylon bags containing both corn silage and corn silage leaf and husk were removed and washed with distilled water until the surfaces of the tissues were cleaned. Nutrient analyses for nutrient digestibility were stated previously following the standard methods published by AOAC International (24).

#### Ruminal VFA Analyses

Ruminal VFA concentrations were measured via high-performance liquid chromatography (Agilent 1100, Santa Clara, CA) to determine formic acid, acetic acid, propionic acid, butyric acid, and lactic acid concentrations following the procedures and chromatographic conditions published by Bai et al. (25).

#### Rumen NH_3_-N Analysis

Ruminal NH_3_-N concentrations were measured following the colorimetric methods as described by Feng and Gao (26) using a 721-type spectrophotometer (Zhengzhou Mingyi Instrument Equipment Co., Ltd., Henan, China). The ruminal NH_3_-N concentration was calculated from the standard curve having an *R*^2^ = 0.9988 (A = 0.3815x – 0.0084), where A is the absorbance, and x is NH_3_-N concentration at 700 nm. The 2-ml rumen fluid sample was diluted with distilled water 5-fold prior to chemical reaction.

### Scanning Electron Microscopy for Silage Tissue

After 48 h of ruminal fermentation and digestion, the nylon bags containing the corn silage tissue samples of leaves and husk were removed, bags opened and residue collected, followed by washing with distilled water repeatedly until the tissue surface was cleaned. Then, the whole sample of the leaf or husk was placed under an SM (Discovery. V 20, Jena, Germany) at 12.6 × magnification for viewing the fiber structure and any cell contents remaining. After completing the observations under the stereomicroscope, samples were subsequently fixed, rinsed, and dehydrated via freeze drying (freeze dryer VFD-21S, Yamato Scientific Co., Ltd., Koto-Ku, Japan) after which a film coating was applied (Sputter coater, MSP-1S, Hitachi High-Technologies, Minto-Ku, Japan). The prepared leaf and husk samples were viewed using an SEM (S 3400 N, Hitachi Science and Technology, Minto-Ku, Japan) to visually observe and record sample cell fiber microstructure at 50 × magnification (23) with photographs taken of each sample from each treatment period for each additive.

### Calculation and Statistical Analyses

The calculation of corn silage ruminal nutrient digestibility (DMD, CPD, NDFD, and ADFD) via *in situ* nylon bag was as follows: nutrient digestibility (%) = 100 × (sample nutrient concentration – residue nutrient concentration)/sample nutrient concentration. Before any statistical analyses were conducted, all data were checked for normality and outliers using the univariate procedure of SAS (version 9.4, SAS Institute Inc., Cary, NC). The box and whisker plots and Shapiro–Wilk test were used to verify that data were normality distributed (*P* > 0.15). All data for the three treatment additives (Co, EO, and EOC) were subjected to least squares ANOVA for a replicated 3 × 3 Latin square design (27) using the PROC MIXED procedure of SAS (version 9.4, SAS Institute Inc., Cary, NC) having three inclusion rates of 0, 4, and 7 g/day. The statistical model used was

(1) $$\begin{array}{l} {\text{Y}}_{\text{ijkl}}=\text{μ}+{\text{I}}_{\text{i}}+{\text{P}}_{\text{j}}+\text{R}\left({\text{S}}_{1}\right)+{\text{S}}_{1}+\left(\text{T }\times \text{ S}\right)+{\text{e}}_{\text{ijkl}} \end{array}$$

where Y_ijlk_ = dependent variable, μ = overall mean, I_i_ = additive inclusion rate, P_j_ = period, R(S_1_) = ram within square, S_1_ = square, (I × S) = inclusion rate by square, and e_ijkl_ = experimental error. If the probability of the interaction of I × S was >0.1, this interaction was eliminated from the model. All sources of variation in the statistical model were considered fixed, except for ram within square, which was considered random.

For the CARR experiment, the statistical model used was:

(2) $$\begin{array}{l} {\text{Y}}_{\text{ijk}}=\text{μ}+{\text{I}}_{\text{i}}+{\text{P}}_{\text{j}}+{\text{R}}_{\text{k}}+{\text{e}}_{\text{ijk}} \end{array}$$

where Y_ijlk_ = dependent variable, μ = overall mean, I_i_ = carrier inclusion rate, P_j_ = period, R_k_ = ram, and e_ijkl_ = experimental error. All sources of variation were considered fixed, except for ram, which was considered random. Significant differences were declared when *P* < 0.05 and trends declared at 0.05 ≤ *P* < 0.10.

## Results and Discussion

### Carrier Impact on Nutrient Digestibilities

Miscommunication/misunderstanding among the coauthors resulted in the need to conduct a later separate experiment evaluating CARR impact on nutrient digestibility and ruminal fermentation. No differences (*P* > 0.10) were observed in nutrient digestibility and ruminal fermentation when rams were fed CARR at 0, 4, and 7 g/day (Table 2), except ruminal NH_3_-N concentrations. Rams fed the CARR at 4 g/day demonstrated a lower ruminal NH_3_-N concentration compared with rams fed CARR at 0 and 7 g/day. Even though ingredients in the CARR could be hypothesized to influence ruminal fermentation and nutrient digestibility, the ingredient inclusion rates and feeding rates of the CARR are insufficient to elicit any beneficial response. These data support the conclusion that the CARR is having little to no impact on ruminal fermentation and nutrient digestibility in the experiments reported below. Benchaar et al. (28) reported that only very high EO doses have demonstrated a response (if observed) in animal performance, digestibility, and ruminal fermentation. Therefore, the conclusions in the following main experiment(s) would be considered valid.

**Table 2:** Effect of adding carrier (CARR) on ruminal nutrient digestibility and ruminal fermentation.

**Measurement**	**CARR-0 g**	**CARR-4 g**	**CARR-7 g**	**SEM**	***P*<^1^**
**Digestibility**					
DM	57.5	57.9	56.6	1.45	0.79
CP	71.1	70.3	68.4	1.88	0.60
NDF	27.1	28.8	26.7	2.80	0.82
ADF	14.4	15.0	15.1	2.97	0.97
**Ruminal parameters**					
pH	6.07	6.20	6.03	0.32	0.90
NH_3_-N, mg/dl	6.1^a^	4.4^b^	5.8^a^	0.38	0.01
Total VFA, mmol/L	95.1	98.6	99.8	19.8	0.99
Acetate, molar %	51.5	52.7	53.0	3.15	0.91
Propionate, molar %	26.6	27.0	26.0	2.84	0.91
Isobutyrate, molar %	1.88	1.34	1.55	0.68	0.67
Butyrate, molar %	12.6	13.3	13.4	4.55	0.98
Isovalerate, molar %	3.28	2.86	2.78	0.65	0.94
Valerate, molar %	3.76	3.10	3.27	0.74	0.74

1 *Probably of F-test for treatment*.

a,b *Means in the same row with differing superscripts differ, P < 0.05*.

### Corn Silage Nutrient Digestibilities

#### Co Feeding Rate

The corn silage ruminal DMD by rams fed 4 and 7 g/day Co was greater (*P* < 0.05) compared with rams fed 0 g/day Co (Table 3). The DMD was increased by 4.51 and 3.01%, respectively, compared with rams fed the control (0 g/day). Increasing the Co feeding rate from 4 to 7 g/day did not (*P* > 0.10) further enhance DMD; thus, sufficient DMD enhancement was achieved by feeding 4 g/day Co. The CPD was 11.4% greater (*P* < 0.05) for rams fed 7 g/day compared with rams fed Co at 0 g/day, while rams fed Co at 4 g/day were 7.8% greater but statistically similar (*P* > 0.10) to rams fed 0 g/day. Measured fiber digestion as NDF and/or HC digestibility was greater (*P* < 0.05) for rams fed 4 and 7 g/day of Co compared with rams fed 0 g/day, while ADF digestion was similar (*P* > 0.10) among treatments. Feeding Co at 4 and 7 g/day enhanced NDF and HC digestion by 11.4 and 7.8%, respectively. The HC fiber fraction would be the more easily digestible fiber fraction compared to the cellulose fraction. These results demonstrate that supplementing Co, as soluble Co lactate (vs. cobalt carbonate), improved the ruminal digestion of DM, CP, NDF, and HC, with a 4 g/day feeding rate appearing to be sufficient for improved digestibility, unless greater CP digestion is warranted, which could be accomplished by increasing the feeding rate to 7 g/day feeding rate (Table 3).

**Table 3:** Effect of adding Co on 48-h ruminal nutrient digestibility of corn silage (%).

**Digestibility**	**Co-0 g**	**Co-4 g**	**Co-7 g**	**SEM**	***P*<^1^**
DM	59.4^b^	63.9^a^	62.4^a^	2.07	0.01
CP	46.4^b^	49.9^b^	57.8^a^	2.51	0.01
NDF	59.4^b^	63.9^a^	62.4^a^	2.07	0.01
ADF	36.3	43.3	31.7	6.50	0.41
Hemicellulose	56.2^b^	63.6^a^	65.9^a^	1.48	0.01

1 *Probably of F-test for treatment*.

a,b *Means in the same row with differing superscripts differ, P < 0.05*.

Previous studies have reported that Co had either significant or numerical improvements for fiber digestion (4, 13, 29). Adding Co to the ration could improve feed digestibility, especially when feeding poor-quality roughages (30). Jiang (31) reported that lactating Holstein cows supplemented with 0.3 mg/kg Co demonstrated improved (*P* < 0.05) cellulose digestibility compared with control. Our results demonstrate that adding 4 g Co lactate resulted in increased NDF and ADF digestibilities of corn silage of 4.5 and 7.01%, respectively, compared with the control (0 g/day) fed rams. These results may be explained by some microbial communities needing more Co and/or because Co forms a crosslink between negatively charged bacteria and negatively charged feeds (32). Zelenak et al. (33) showed that the supplementary feeding of Co to goats significantly increased hay digestibility. Feeding Co also improved corn silage DMD and CPD in this study, which was similar to results in the cattle (34), where authors reported that Co improved cattle BW gains when feeding non-leguminous hay combined with urea improved cellulose digestion. Hatfield (35) cited work that proteins are frequently associated with lignin–carbohydrates complexes, and the speculation is that enhance fiber digestion results in greater CP digestion.

In our study, NDFD and HCD increased with 4 g of Co, while ADFD was similar among all Co inclusion rates, demonstrating that supplementing 4 g was optimal for fiber degradation. These results are similar to the results reported by Liu et al. (36) in rabbits demonstrating that daily gain and feed efficiency were improved with a moderate Co inclusion rate, but a larger Co inclusion rate resulted in poorer BW gain and feed efficiency. Other studies confirmed that a specific Co amount in the ration can maintain the amount and type of rumen bacteria and parasites at normal levels, but excess Co can hinder their growth (37). Therefore, Co supplementation requires careful control ration inclusion rates. A large number of experimental studies have suggested that supplementing 5 mg/kg Co chloride (soluble like Co lactate) to sheep daily can improve ruminal fiber digestibility (38). Co concentrations above 0.50 mg/kg were appropriate for goats (39), whereas a concentration of 0.25 mg/kg is adequate for sheep (40). In this study, feeding 4 g Co lactate was adequate without being excessive.

#### EO Feeding Rate

Ruminal corn silage DM, CP, and NDF digestibilities were similar (*P* > 0.10) among rams fed all treatments (Table 4). Corn silage HC digestibility was 7.3% greater (*P* < 0.05) for rams fed EO at 4 g/day compared with rams fed 0 and 7 g/day. Rams fed 7 g/day of EO was similar (*P* > 0.10) compared with rams fed 0 g/day (control) but numerically improved 5.0%, which indicates that more animals were needed to become significant.

**Table 4:** Effect of adding essential oils (EO) on 48-h ruminal nutrient digestibility of corn silage (%).

**Digestibility**	**EO-0 g**	**EO-4 g**	**EO-7 g**	**SE**	***P*<^1^**
DM	55.3	56.0	54.9	3.57	0.98
CP	46.5	49.1	44.5	5.42	0.67
NDF	38.0	36.6	36.5	4.75	0.96
ADF	28.5	27.2	30.1	6.22	0.89
Hemicellulose	64.1^b^	71.4^a^	69.1^ab^	1.28	0.01

1 *Probably of F-test for treatment*.

a,b *Means within same row with differing superscripts differ, P < 0.05*.

In this study, the corn silage ruminal DM, CP, NDF, and ADF digestibility demonstrated no influences by EO or increasing EO feeding rates but was numerically increased by 0.65 and 2.58%, respectively, when supplemented with 4 g/day EO compared with the 0 g/day EOC (control). Oregano essential oil increased nutrient digestibility and milk protein concentration by dairy cows (41), which is consistent with proposed EO mechanisms. Several EO are known to stimulate appetite, activate digestive enzymes through biofeedback, change chyme viscosity, and increase feed intake (42, 43). However, ruminal nutrient digestibilities were similar with increasing EO feeding rates in this study using sheep, which could potentially be explained by the test animal species, EO feeding rate, and/or different metabolic mechanisms (i.e., ruminal vs. postruminal) by different species. Thao et al. (22) reported similar nutrient (DM, CP, NDF, and ADF) digestibilities when feeding Eucalyptus oil to water buffalos. Froehlich et al. (7) suggested that in the past, EO may have been fed at too high of a feeding rate, thereby preventing performance improvements.

#### EOC Feeding Rate

The corn silage ruminal NDF and ADF digestibilities were greater (*P* < 0.05) for rams fed the EOC combination at 4 and 7 g/day compared with rams fed 0 g/day (control; Table 5). The DM, CP, and HC digestibilities were similar (*P* > 0.10) among rams receiving all treatments. Feeding the EOC combination above 4 g/day resulted in little improvement in ruminal nutrient digestibility.

**Table 5:** Effect of adding a cobalt and essential combination (EOC) on 48-h ruminal nutrient digestibility of corn silage (%).

**Digestibility**	**EOC-0 g**	**EOC-4 g**	**EOC-7 g**	**SE**	***P*<^1^**
DM	57.7	60.0	60.0	2.85	0.20
CP	38.3	36.8	37.6	6.01	0.99
NDF	21.4^b^	28.8^a^	27.7^a^	5.02	0.01
ADF	24.3^b^	33.3^a^	34.4^a^	5.74	0.02
Hemicellulose	70.9	73.5	72.6	0.88	0.22

1 *Probably of F-test for treatment*.

a,b *Means within the same row with differing superscripts differ, P < 0.05*.

Studies have shown that EOC is beneficial to ruminants consuming high-fiber diets. The results indicated that the fiber digestibility increased by 12–22% when using EOC at Oklahoma State University (unpublished). Kuester (44) found that the roughage digestibility of DM, CP, NDF, and ADF were improved by 2.3, 3.5, 3.8, and 4.2%, respectively, by lactating dairy cows fed the same EOC product at 28 g/day, compared with lactating cows fed 0 g/day EOC (control), similar to this experimental conclusion, where the DMD improved by 2.29 and 2.33% when sheep were fed 4 and 7 g EOC. The NDFD and ADFD improved by 6.30–7.39 and 9.05–10.12%, respectively (*P* < 0.05). As noted above, oregano EO are natural feed additives. Oregano EO can alter the intestinal microbial community structure and optimize the animal gut microflora environment (13). Cobalt is one of the indispensable trace elements and plays a crucial role in crude fiber digestion. Both are present in EOC and play coordinated roles to improve the rumen environment, enhancing the activity of cellulolytic bacteria to promote the rumen fermentation functions and forage utilization. For CPD, NDFD, ADFD, and HCD, there were no significant differences between 4 and 7 g (*P* > 0.05), which suggested that a 4-g EOC inclusion rate is adequate for sheep.

Based on the current evaluation of different additives, we found that adding EO alone could enhance ruminal HC digestion, which would be in agreement with Thao et al. (22). Thao et al. (22) reported improved NDF digestibility with similar ADF digestibility, which indicates that HC digestibility was improved with EO. Co significantly improved the corn silage ruminal DM, NDF, and HC digestibility, illustrating that Co in combination with EOC probably played an important role in straw fiber digestion and degradation; however, EO may still be involved in the maintenance of the rumen microbial flora. There is at least a numerical indication of synergism between Co and EO (oregano). If it is assumed that the oregano EO alone has a small negative influence on NDFD and ADFD (−1.4 and −1.3%, respectively) and Co alone improved NDFD and ADFD by 4.5 and 7.0%, respectively, then it could be expected that the combination would result in improvements in NDFD and ADFD of 3.1 and 5.7%, respectively, assuming no synergism or antagonism. However, supplementation of the EO and Co combination (EOC) resulted in NDFD and ADFD improvements of 7.4 and 9.1%, respectively. While not definitive, this does suggest the need for further research into mechanisms involved in the activities of oregano EO and Co and their possible synergism related to ruminal fiber digestion by ruminants.

### Ruminal Fermentation

#### Co Feeding Rate

Ruminal pH was similar (*P* > 0.10) for rams fed all Co inclusion rates (Table 6). In contrast, rams fed Co at 7 g/day demonstrated greater (*P* < 0.05) ruminal NH3–N, total VFA, and acetate concentrations compared with rams fed 0 and 4 g Co/day. Concentrations of propionate, butyrate, and the acetate to propionate ratio were similar (*P* > 0.10) among rams fed all Co inclusion rates. The CPD increase observed in Table 3 may probably be associated with the increase in ruminal NH3–N concentrations, while the increases in DM, NDF, and HC digestibilities may be influencing increases in total VFA and acetate concentrations. Greater fiber digestion would be expected to increase ruminal acetate concentrations.

**Table 6:** Effect of Co feeding rate on sheep ruminal characteristics.

	**Feeding rate of Co, g/day**				
**Measurement**	**0**	**4**	**7**	**SEM**	***P*<^1^**
pH	6.37	6.34	6.16	0.07	0.20
NH_3_-N, mg/dl	9.99^b^	11.1^b^	15.8^a^	0.72	0.01
**VFA, mmol/L**					
Total	62.2^b^	59.4^b^	71.7^a^	0.75	0.01
Acetate	47.7^b^	47.7^b^	55.3^a^	1.24	0.01
Propionate	12.0	11.7	11.3	0.46	0.61
Butyrate	5.14	5.15	5.20	0.10	0.39
**Acetate/propionate ratio**					
	3.98	4.07	4.89	0.40	0.11

1 *Probably of F-test for treatment*.

a,b *Means within the same row with different superscripts differ, P < 0.05*.

#### EO Feeding Rate

Rams fed EO at 7 g/day had greater (P < 0.05) ruminal pH compared with rams fed 0 and 4 g/day (Table 7). Ruminal NH3–N, total, and individual VFA concentrations were similar (*P* > 0.10) among rams fed all EO inclusion rates. Even though increasing EO inclusion rate increased HC digestibility (Table 4), the increased HC digestibility demonstrated little influence on ruminal fermentation parameters, except pH (Table 7). In agreement, Thao et al. (22) reported Eucalyptus having no effect on ruminal pH and NH3–N concentrations.

**Table 7:** Effect of essential oils (EOs) feeding rate on sheep ruminal characteristics.

	**Feeding rate of EO, g/day**				
**Measurement**	**0**	**4**	**7**	**SEM**	***P*<^1^**
pH	6.35^b^	6.29^b^	6.53^a^	0.04	0.03
NH_3_-N, mg/dl	14.2	14.0	11.3	0.91	0.15
**VFA, mmol/L**					
Total	53.1	64.7	65.7	4.03	0.16
Acetate	48.1	49.2	49.1	1.24	0.65
Propionate	14.4	14.7	15.0	2.23	0.16
Butyrate	5.07	5.43	4.95	0.80	0.65
**Acetate/propionate ratio**					
	3.44	3.60	3.28	0.88	0.91

1 *Probably of F-test for treatment*.

a,b *Means within the same row with different superscripts differ, P < 0.05*.

#### EOC Feeding Rate

Feeding 4 and 7 g/day of the EOC combination resulted in rams having a lower (*P* < 0.05) ruminal pH than rams fed EOC at 0 g/day (Table 8). Feeding a combination of Co and EO (EOC) influences ruminal pH in contrast to feeding the individual Co and EO components. Ruminal NH3–N concentrations were similar (*P* > 0.10) among rams fed all inclusion rates, while total VFA concentrations were lower (*P* < 0.05) for rams fed EOC at 7 g/day compared with rams fed 0 and 4 g/day. Rams fed EOC at 4 g/day had greater acetate concentrations than rams fed 0 g/day, while rams fed EOC 7 g/day had the lowest (*P* < 0.05) acetate concentrations compared with rams fed EOC at 0 and 4 g/day. No differences (*P* > 0.10) were observed for propionate, butyrate, and acetate to propionate ratios.

**Table 8:** Effect of essential oils and cobalt combination (EOC) feeding rate on ruminal characteristics of sheep.

	**Feeding rate of EOC, g/day**				
**Measurement**	**0**	**4**	**7**	**SEM**	***P*<^1^**
pH	6.00^a^	5.82^b^	5.77^b^	0.04	0.01
NH_3_-N, mg/dl	15.4^a^	15.0^a^	14.4^b^	0.41	0.29
**VFA, mmol/L**					
Total	64.8^a^	64.9^a^	60.9^b^	1.28	0.04
Acetate	52.4^b^	54.0^a^	48.5^c^	0.15	0.01
Propionate	10.9	10.9	10.6	0.14	0.13
Butyrate	4.71	4.96	5.33	0.41	0.62
**Acetate/propionate ratio**					
	4.83	4.96	4.58	1.08	0.22

1 *Probably of F-test for treatment*.

a,b,c *Means within the same row with different superscripts differ, P < 0.05*.

Rumen fluid pH can be a direct ruminal fermentation rate indicator, which usually changes rapidly with feeding time and feed types, ranging from ~5.0 to 7.5 (45). In this experiment, ruminal pH was lowered when rams were fed the EOC combination compared to rams fed the 0 g/day EOC (control; *P* < 0.05). However, ruminal pH of all additives evaluated in this study at different inclusion rates were within normal ranges, indicating a normal functioning ruminal environment. These results suggest that feeding the EOC combination could be implemented compared with Co or EO independently to effectively reduce ruminal pH, thereby increasing bacteriostatic efficacy without incurring acidosis.

Wang (46) found that adding 0.1 mg/kg of Co increased artificial rumen pH by 0.32%, but pH of the Co treatment was similar to the control (*P* > 0.05). With the addition of 1.0 mg/kg of Co, the pH rose to 6.31, greater than the control by 0.96% (*P* < 0.05), which demonstrated that ruminal pH rose with increased Co. However, Miao (47) found that pH had a tendency to decrease with the addition of different levels of Co in an *in vitro* experiment using an artificial rumen (*P* > 0.05), which is similar to the present results. In summary, regardless of Co inclusion rate, the average ruminal pH was in the normal range, which was advantageous to fiber digestion, in addition to nutrient fermentation and rumen microbial growth.

The NH_3_-N concentration can reflect the feed N content, N solubility, and degradation rate along with intake of these components. Generally, the ruminal NH_3_-N concentration range is 10–50 mg/dl. This value will usually peak ~1.5–1 h after feeding (48). The NH_3_-N concentration changed in the range of 9.99–15.8 mg/100 ml under the three additives and their inclusion rates, indicating that each feed additive did not alter rumen NH_3_-N beyond normal limits. The NH_3_-N concentrations increased with increasing Co inclusion rate that was different from increasing EOC and EO inclusion rates. Co is a vitamin B_12_ component, which plays an important role in animal protein metabolism (40). The Co may have provided positive ruminal fermentation regulation by being beneficial to rumen microbial activity (47, 48). Feeding EO and EOC numerically reduced NH_3_-N concentrations, but these differences were not significant (*P* > 0.05), which may be due to EO promoting the growth and proliferation of rumen microbes (40, 49). Thymol in EO will act on rumen microorganisms and affect amino acid deamination (50, 51). All of these results indicated that EO and EOC could potentially impact ruminal NH_3_-N concentrations, but Thao et al. (22) reported no impact on NH_3_-N concentrations.

Ruminal VFA concentrations reflect microbial activity, ruminal absorption, and/or ruminal passage rates (48). Increasing Co inclusion rates influenced TVFA and acetic acid (*P* < 0.05) but demonstrated little impact on propionic acid (*P* > 0.05) concentrations. The TVFA and acetic acid concentrations were greater for rams fed 7 g/day than rams fed 0 (control) and 4 g/day inclusion rates (*P* < 0.05). This difference is likely due to Co being a critical trace mineral for rumen microbial activity. Some microbes can use Co to synthesize vitamin B_12_ and other rumen microbial growth factors (52). Vitamin B_12_ synthesis is necessary for many bacterial enzymes, and these enzymes participate propionic acid metabolism along with synthesis of methane, acetic acid, and methionine (3, 53). Research using diets comprised mainly of wheat straw demonstrated that increasing Co inclusion rates significantly increase TVFA and acetic acid, propionic acid, and butyric acid production (*P* < 0.05; 27). However, Hussein et al. (29) and Tiffany et al. (54) concluded that there was no effect on fermented liquid TVFA using *in vitro* culture condition, which may be related to the animal species, test diet, trial duration, and/or other factors. In contrast, Thao et al. (22) reported that Eucalyptus oil decreased ruminal acetate and increases ruminal propionate concentrations in water buffalos, which may indicate that varying the EO will have varying impacts on ruminal fermentation.

In general, increasing Co inclusion rates increased TVFA and acetic acid concentrations, while EO (oregano) demonstrated minimal impacts for VFA, and finally, the EOC combination decreased TVFA and acetic acid at increasing inclusion rates, i.e., 7 g/day. It could be possible for a synergistic effect between Co and EO (EOC combination) improved rumen wall VFA absorption to maintain normal ruminal metabolic function, causing VFA concentrations to decline. This speculation needs to be evaluated in future research.

### SEM Scans of Corn Silage Husk and Leaf Fiber Structures After Ruminal Digestion

**Figure 1:**
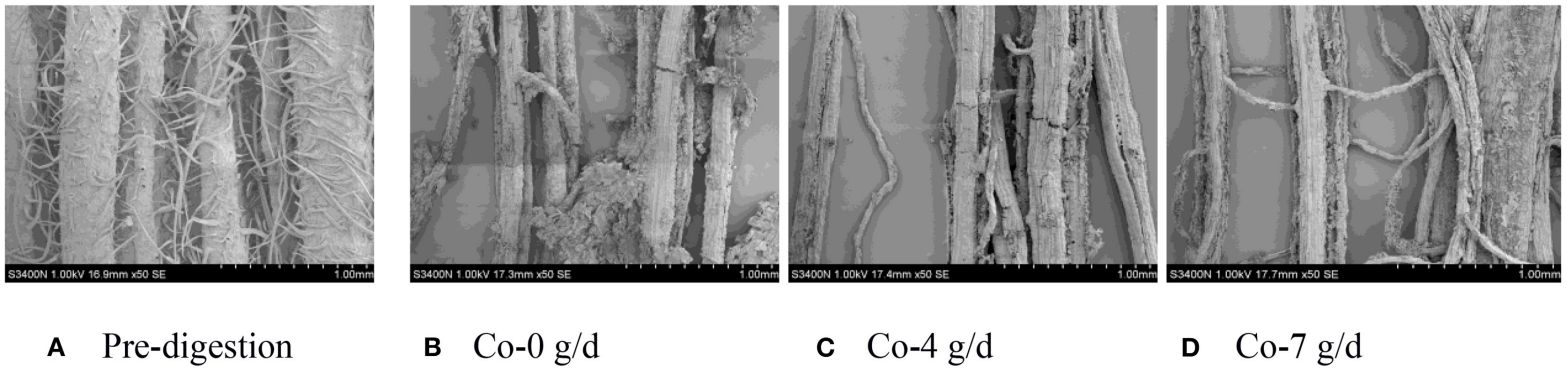
**(A–D)** Scanning electron microscope (SEM) images at 50 × magnification demonstrating corn silage husk degradation and remaining fiber structure when rams were fed cobalt at 0 (Co-0), 4 (Co-4), or 7 g/day (Co-7).

**Figure 2:**
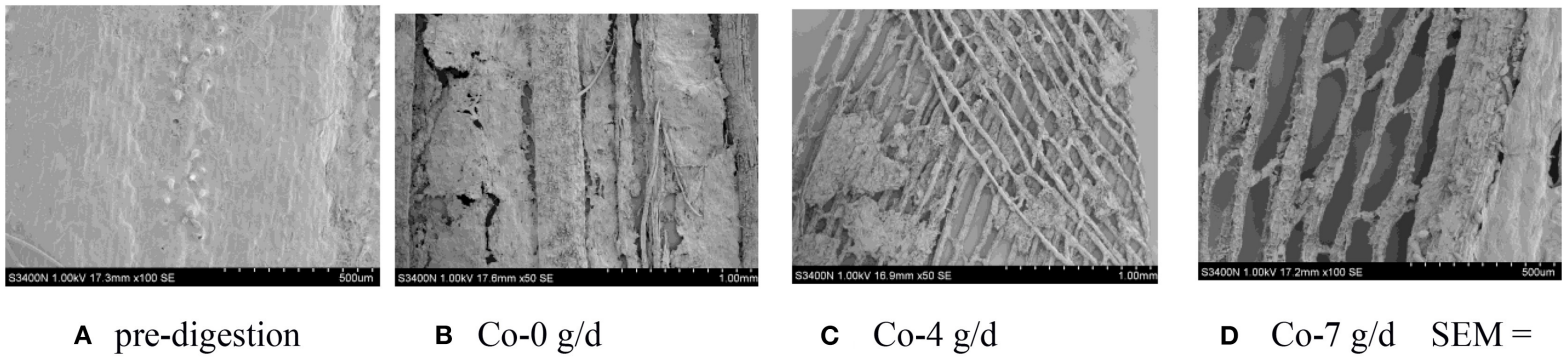
**(A–D)** Scanning electron microscope (SEM) images at 50 × magnification demonstrating corn silage leaf degradation and remaining fiber structure when rams were fed cobalt at 0 (Co-0), 4 (Co-4), or 7 g/day (Co-7).

**Figure 3:**
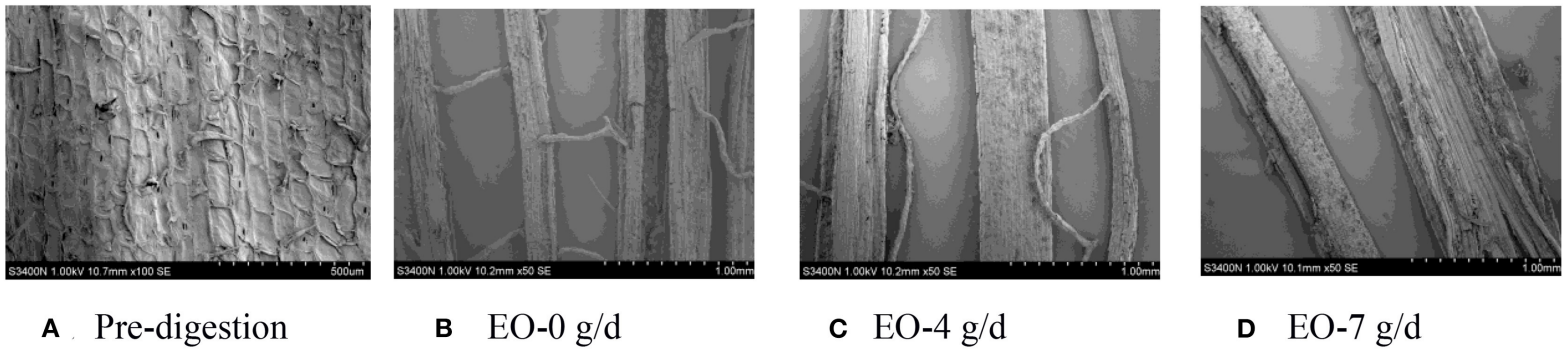
**(A–D)** Scanning electron microscope (SEM) images at 50 × magnification demonstrating corn silage husk degradation and remaining fiber structure when rams were fed essential oils (EOs) at 0 (EO-0), 4 (EO-4), or 7 g/day (EO-7).

**Figure 4:**
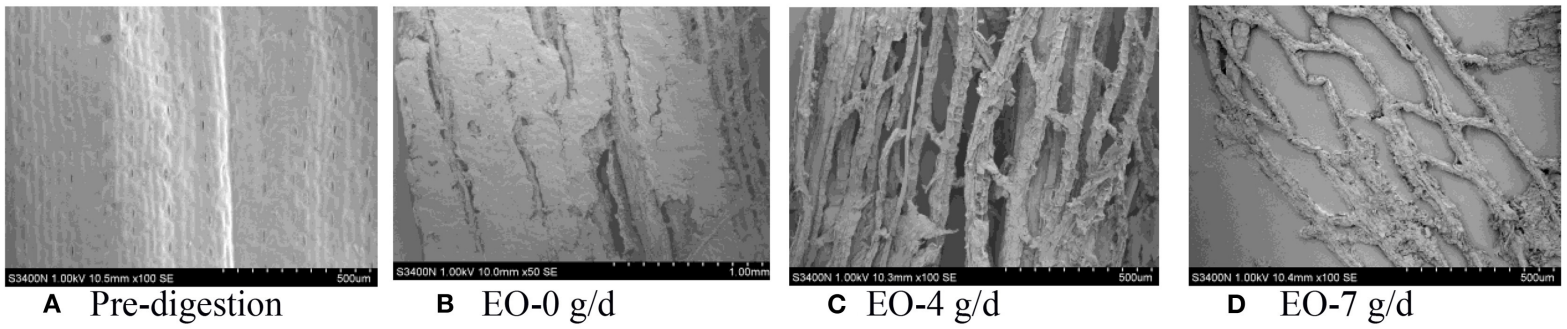
**(A–D)** Scanning electron microscope (SEM) images at 50 × magnification demonstrating corn silage leaf degradation and remaining fiber structure when rams were fed essential oils (EOs) at 0 (EO-0), 4 (EO-4), or 7 g/day (EO-7).

**Figure 5:**
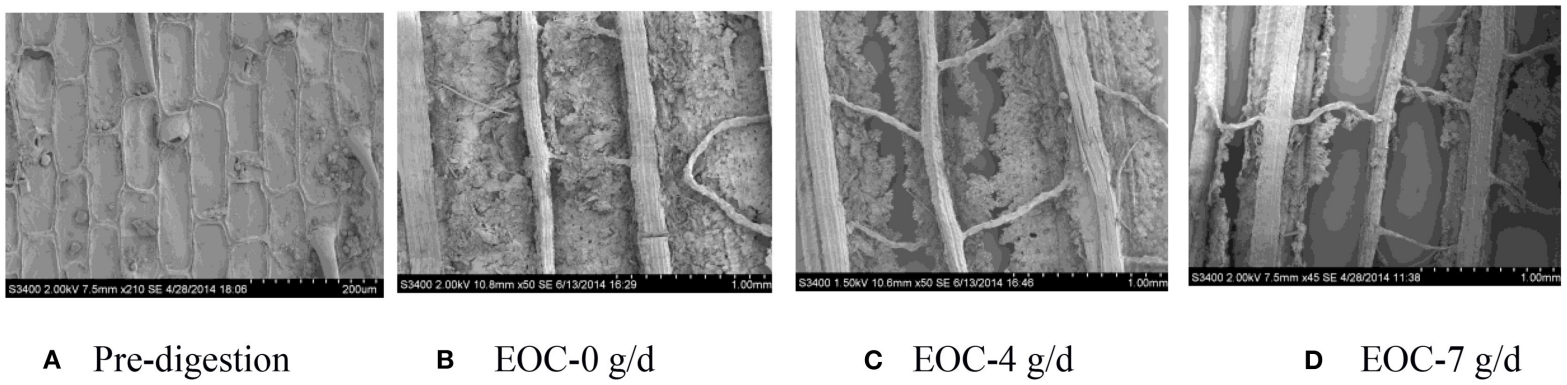
**(A–D)** Scanning electron microscope (SEM) images at 50 × magnification demonstrating corn silage husk degradation and remaining fiber structure when rams were fed cobalt and essential oils (EOCs) at 0 (EOC-0), 4 (EOC-4), or 7 g/day (EOC-7).

**Figure 6:**
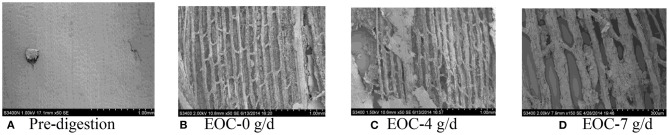
**(A–D)** Scanning electron microscope (SEM) images at 50 × magnification demonstrating corn silage leaf degradation and remaining fiber structure when rams were fed cobalt and essential oils (EOCs) at 0 (EOC-0), 4 (EOC-4), or 7 g/day (EOC-7).

**Figure 7:**
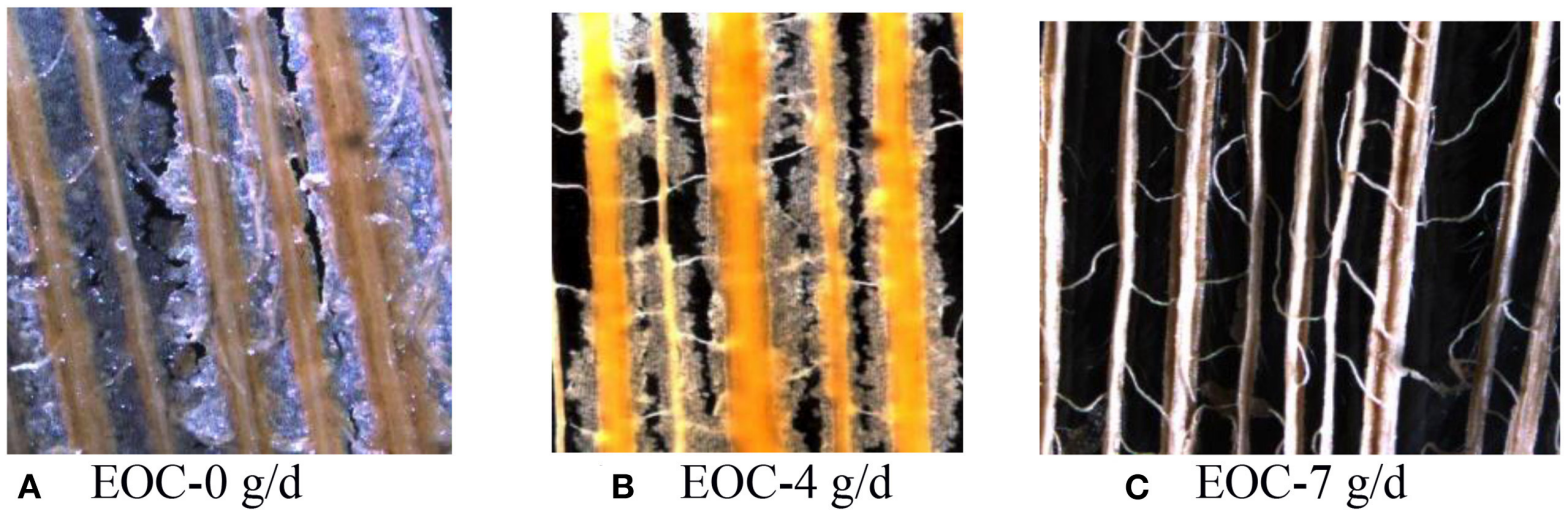
**(A–C)** Stereo microscopy (SM) scans at 12.6 × magnification demonstrating corn silage husk degradation and remaining fiber structure when rams were fed cobalt and essentials (EOCs) fed at 0 (EOC-0), 4 (EOC-4), or 7 g/day (EOC-7).

**Figure 8:**
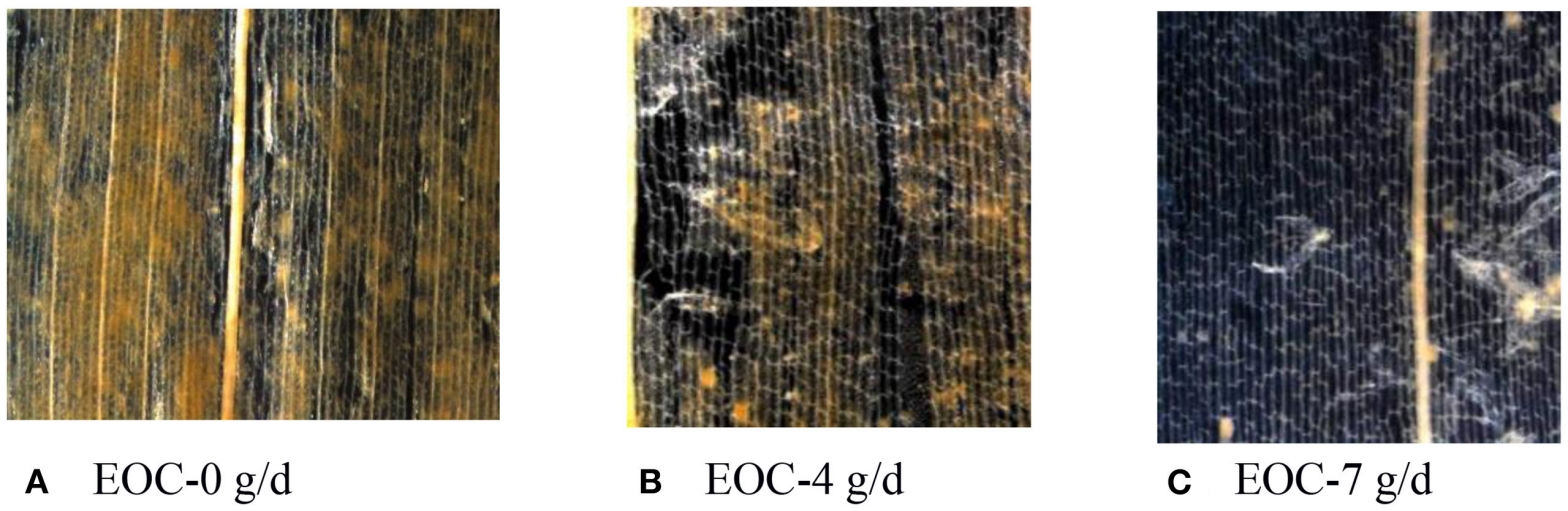
**(A–C)** Stereo microscopy (SM) scans at 12.6 × magnification demonstrating corn silage leaf degradation and remaining fiber structure when rams were fed cobalt and essentials (EOCs) fed at 0 (EOC-0), 4 (EOC-4), or 7 g/day (EOC-7).

The corn silage husks and leaves were incubated in the rams using nylon bags for ruminal *in situ* degradation. Each figure (Figures 1–8) contains micrographs of a sample group comprised of a preruminal digestion scan of the raw material and then scanned after 48 h of ruminal *in situ* degradation with increasing additive inclusion rate. These scans were visually appraised because, to our knowledge, no mechanism exists, nor could we find or develop one, to convert these scans into a measurement (absorbance, area, volume, etc.) that could be statistically evaluated. Therefore, the SEM and SM scans on plant cell structure were visually evaluated and used to confirm the measured digestibility parameters presented in the preceding tables. In addition, the ruminal *in situ* samples for each additive (Co, EO, and EOC) from each sampling period were visually appraised and found to be fairly consistent (minimal visual variation) across each ram for each period. Thus, the figures containing the scans are presented as being representative of the entire additive across periods at the increasing inclusion rates for that experiment.

#### Co Feeding Rate

The corn silage husk samples visually demonstrated dense epidermal trichomes and integrated skin before ruminal digestion (Figure 1A), while increasing Co inclusion rate visually demonstrated increasing surface area digestion by ruminal microbes when rams were fed Co at 4 and 7 g/day compared with rams fed Co at 0 g/day (Figure 1D). For rams fed increasing Co inclusion rates at 4 and 7 g/day, the trichomes and mesophyll were completely digested leaving only disarrayed parallel veins compared to rams fed Co at 0 g/day, which demonstrated only partial digestion.

The corn silage leaf ruminally incubated *in situ* visually demonstrated a neatly arranged epidermal cells with wax, epidermal trichomes, guard cells, closed stomas, whole mesophyll, and vein structure (Figure 2A). Compared with the ruminally undigested material, feeding Co at 0 g/day demonstrated visual disappearance of epidermal trichomes and broken surfaces with partially digested mesophyll. However, by increasing Co inclusion rates to 4 and 7 g/day fed to the rams, the ruminal degradation demonstrated a visually complete digestion of leaf epidermal cells, palisade tissue, and spongy tissue leaving only the netted veins (Figures 2B–D).

#### EO Feeding Rate

Prior to ruminal *in situ* degradation, the corn silage husks cells were neatly arranged, with clear cell walls, completed cell frames, and visible epidermal trichomes with veiny and hairy stomas (Figure 3A). Compared with the preruminal degradation husk, rams fed all EO inclusion rates demonstrated that the corn silage husk was completely digested, leaving only the epidermal cells, palisade tissue, or spongy tissue, with smooth and neatly arrayed parallel veins (Figures 3B–D).

Similar to the corn silage husks, the preruminal *in situ* degradation of the corn silage leaf epidermal cells were neatly arranged with clear cell walls and complete cell frames, along with visible epidermal trichomes and veiny and hairy stomas (Figure 4A). Compared to the preruminal degradation, rams fed 0 g/d EO demonstrated that the plant cell wax layer was damaged, leaving the palisade and spongy tissues outside; the mesophyll was partially destroyed, with complete veins, but no epidermal trichomes (Figure 4B). Rams fed 4 g/day EO demonstrated that mesophyll was partially degraded after 48 h (Figure 4C) compared to rams fed EO at 0 g/day, while the rams fed 7 g/d EO demonstrated that the mesophyll was completely degraded with only some thin and clear reticulate veins remaining (Figure 4D).

#### EOC Feeding Rate

The preruminal *in sit*u degradation of the corn silage husk was as described previously (Figure 5A). Feeding rams 0 g/day EOC (the combination of Co and EO) demonstrated damaged husk epidermal wax and mesophyll with a clear cell wall (Figure 5B) compared to the preruminal sample. Compared to rams fed 0 g/d EOC, feeding 4 g/day EOC demonstrated a partially digested palisade tissue and mesophyll (Figure 5C), compared with feeding 7 g/day EOC, which demonstrated a more complete husk degradation with only veins remaining visual (Figure 5D).

The preruminal *in situ* degradation of the corn silage leaf material was as described previously (Figure 6A). Feeding rams 0 g/day EOC after 48 h of ruminal degradation demonstrated a visually damaged wax layer with some epidermal cell degradation compared with the preruminal sample (Figure 6B). Compared to rams fed 0 g/day EOC, rams fed 4 g/day EOC demonstrated the leaf epidermal cells being completely digested with leaving only spongy tissue outside, while degrading part of the mesophyll (Figure 6C). Rams fed 7 g/day EOC demonstrated a complete leaf degradation compared with rams fed lower EOC inclusion rates (Figure 6D).

### Stereomicroscope Scans After Ruminal Digestion

The effort required to process samples for SM scanning along with our major interest in the EOC combination performance resulted in selecting only samples from rams fed increasing EOC inclusion rates for SM scanning at 12.5 × magnification. Feeding rams increasing EOC inclusion rates visually demonstrated increasing corn silage husk degradation (Figures 7A–C), with rams fed 7 g/day EOC demonstrating remaining husks containing only clear mesh veins. Corn silage leaves demonstrated the same increasing degradation with increasing EOC inclusion rates with rams fed 7 g/day EOC in which the leaf residue was completely transparent with only the vein structure remaining (Figures 8A–C).

Previous Co and EO studies have focused on the feeding responses as measured by animal production performance, nutrient utilization, and livestock immune function (7, 17, 44). To our knowledge, there are no scientific literature studies, and this is the first time that SEM and SM scans were used to study sheep ruminal straw cell fiber degradation in the absence or presence of different feed additives. While quantification would be preferred, future studies may be able to develop procedures to quantify ruminal digestion using SEM and SM techniques. However, in this study, SEM and SM techniques provided a direct visual demonstration to supplement and compliment ruminal digestibility measurements for the first time.

Plant cell walls and fibrous components of cellulose, hemicellulose, and lignin are usually the most difficult material for ruminant animals to degraded (55). Straw plant surfaces are covered with a wax layer and cuticle, which is difficult to be rapidly degraded by ruminal microbes and enzymatic degradation. Ruminal microbes depend mainly on a damaged surface and stoma to initiate degradation and digestion. Therefore, cells located in the inner layer are digested later because the plant cells are tightly arranged with overlapping cell walls (56). Thus, rupturing the plant cell wall will expose cell contents, which is critical for feed degradation, nutrient digestion, and feed additive effectiveness.

In this study, when sheep were fed 4 and 7 g/day Co, the epidermal cells, palisade tissue, and spongy tissue of corn silage leaves were completely degraded, with only net-like veins remaining compared with rams fed 0 g/day Co. The corn husk exhibited similar degradation changes under the same conditions with the leaf mesophyll between veins disappearing completely with disorganized parallel veins remaining when rams were fed 4 and 7 g/day. These observations support the conclusion that Co is a microbial catalyst that can improve ruminal microbial activity, especially for cellulosic microbes. Improving ruminal microbial cellulosic activity will enhance fiber digestibility of straw (3, 30, 31, 37, 40, 57, 58).

When feeding increasing EO inclusion rates to rams, the ruminal degradation of the corn silage husk and leaf resulted in morphological changes similar to those observed with feeding increasing Co inclusion rates. The speculation is that plant phenolic compounds maintained the ruminal microbial system balance for the combination of ruminal bacteria, protozoa, and fungi for completing plant cell wall degradation (56).

Feeding increasing EOC inclusion rates demonstrated increased ruminal leaf degradation, but an apparent synergistic effect did not occur when combining Co lactate and EO on fiber degradation. The apparent lack of synergism may be due to each additive's independent efficacy to facilitate fiber digestion. Oregano EO is known to exert bacteriostatic efficacy, which may maintain ruminal microbial balance to ensure a healthy rumen with normal fermentation. The soluble Co lactate provides an essential trace element for maintaining gut biome health. With each demonstrating slightly different mechanisms, a combination of Co and EO would ensure greater degradation of straw and improve feed nutrient and fiber digestibility.

## Conclusions

These results suggested that the optimal feeding rate would be 4 g/day of Co or EO, which would be ~27 g/day for large frame ruminants (i.e., 650 kg). The integration of digestibility estimates with SEM and SM micrographs of digested forage particles can be beneficial in visually confirming nutrient digestibility estimates. Corn silage husk has a high NDF and HC content that is usually very digestible, as visually confirmed by the SEM. Corn silage husk is composed mainly of xylose, which is a highly digestible sugar compared to arabinose. In contrast, corn silage leaves are high in NDF and ADF concentrations, resulting in a high cellulose content, which has much greater variation in the observed digestibility, suggesting that feed additives, such as oregano EO and Co, could enhance cellulose digestibility. The use of SEM and SM may be beneficial when evaluating nutrient digestibility technologies to enhance ruminal nutrient digestion.

## Data Availability Statement

The raw data supporting the conclusions of this article will be made available by the authors, without undue reservation.

## Author Contributions

TJ was responsible for the trial implementation, supervision of students collecting and analyzing samples, and manuscript preparation. JW is the overall project leader providing financial support and experimental conception. DC was involved in data analyses, statistical analyses, language revisions, journal selection, and manuscript submissions and revisions. DD, MB, and BH contributed to experimental design and providing additives evaluated in the study. SZ contributed to the supervision and assistance of students in managing animals, collecting, and analyzing samples. JL was an undergraduate student assisting with sample analyses, scanning electron microscope, and data collection and organization. ZL contributed to supervision of additive inclusion, sample collection and analysis, and manuscript editing. All authors contributed to the article and approved the submitted version.

## Conflict of Interest

BH and DD are employed by Ralco, Inc., while DC is a paid outside consultant by Ralco, Inc and is employed by Casper's Calf Ranch, LLC. MB was an outside paid consultant by Gansu Agricultural University. The remaining authors declare that the research was conducted in the absence of any commercial or financial relationships that could be construed as a potential conflict of interest.

## Abbreviations

Co: cobalt lactate
EO: essential oils
EOC: essential oils and cobalt
DM: dry matter
CP: crude protein
NDF: neutral detergent fiber
ADF: acid detergent fiber
HC: hemicellulose
SEM: scanning electron microscopy
SM: steroscopic microscopy
FDA: Food and Drug Administration
VFA: volatile fatty acids
TVFA: total volatile fatty acids
CARR: carrier
TMR: total mixed ration
NH_3_-N: ammonia nitrogen
DMD: dry matter digestibility
CPD: crude protein digestibility
NDFD: neutral detergent fiber digestibility
ADFD: acid detergent fiber digestibility
Y_ijk_: dependent variable
T_i_: treatment
P_j_: period
R_k_: ram
e_ijk_: radom error.
